# Postoperative hypothermia following non-cardiac high-risk surgery: A prospective study of temporal patterns and risk factors

**DOI:** 10.1371/journal.pone.0259789

**Published:** 2021-11-15

**Authors:** Itajiba Paternosti Sabbag, Fabio Barlem Hohmann, Murillo Santucci Cesar Assunção, Renato Carneiro de Freitas Chaves, Thiago Domingos Corrêa, Pedro Ferro L. Menezes, Ary Serpa Neto, Luiz Marcelo Sá Malbouisson, Suzana Margareth Ajeje Lobo, Cristina Prata Amendola, Jose Eduardo de Aguilar-Nascimento, João Manoel Silva, on behalf of The BRASIS Study Group

**Affiliations:** 1 Hospital das Clínicas, Medical School, Universidade de São Paulo, São Paulo, SP, Brazil; 2 Departamento de Pacientes Graves, Hospital Israelita Albert Einstein, São Paulo, SP, Brazil; 3 Unidade de Terapia Intensiva, Hospital de Base, Medical School of São José do Rio Preto, São José do Rio Preto, SP, Brazil; 4 Unidade de Terapia Intensiva, Hospital de Câncer de Barretos, Pio XII Foundation, Barretos, SP, Brazil; 5 Unidade de Terapia Intensiva, Universidade Federal de Mato Grosso, Cuiabá, MT, Brazil; Ohio State University Wexner Medical Center Department of Surgery, UNITED STATES

## Abstract

**Background and objectives:**

Hypothermia occurs commonly during surgery and can cause postoperative complications. We aimed to describe the characteristics and outcomes of hypothermia in patients undergoing major surgeries.

**Methods:**

This prospective, observational, multicenter study of a nationally representative sample included all patients over 18 years of age admitted to an intensive care unit (ICU). Thirty ICUs were selected randomly at national level. The main outcome measure was the proportion of patients who developed postoperative hypothermia in the first 24 hours of ICU admission. Patients were divided into three groups based on temperature: <35°C, <36°C, and ≥36°C (no hypothermia). Patients’ characteristics, postoperative complications, and risk factors were evaluated in all groups. To verify whether hypothermia was a strong risk factor for postoperative complications, a Kaplan–Meier curve was generated and adjusted using a Cox regression model.

**Results:**

In total, 738 patients had their temperatures measured. The percentage of patients with temperature <35°C (median [Q1-Q3], 34.7°C [34.3–34.9°C]) was 19.1% (95% confidence interval [CI] = 16.1–22.5) and that of patients with temperature <36°C (median [Q1-Q3], 35.4°C [35.0–35.8°C]) was 64% (95% CI = 58.3–70.0). The percentage of surgical complications was 38.9%. Patients with hypothermia were older, had undergone abdominal surgeries, had undergone procedures of longer duration, and had more comorbidities. A postoperative temperature ≤35°C was an independent risk for composite postoperative complications (hazard ratio = 1.523, 95% CI = 1.15–2.0), especially coagulation and infection.

**Conclusions:**

Inadvertent hypothermia was frequent among patients admitted to the ICU and occurred more likely after abdominal surgery, after a long procedure, in elderly patients, and in patients with a higher number of comorbidities. Low postoperative temperature was associated with postoperative complications.

## Introduction

Body temperature is an important vital sign and should be monitored in the perioperative period, as hypothermia can cause postoperative complications [1, 2]. In general, the average body temperature ranges from 36.7°C to 37°C, with variations of 0.2°C to 0.4°C; unintentional hypothermia is defined as a body temperature below 36°C [3, 4].

Hypothermia is a common event that affects more than 70% of patients due to direct inhibition of thermoregulation by anesthetics, decreased metabolism, and exposure of the patient to the surgical environment, which usually ranges from 18°C to 23°C [5]. Regard to the thermal discomfort caused, hypothermia is also associated with complications, such as cardiac arrhythmia, increased mortality, increased infection of surgical wounds, and intraoperative coagulation dysfunction, with consequent increase in the number of transfusions in the postoperative period, in the post-anesthesia care unit, and at the hospital. These complications, in addition to being harmful to patients, result in higher costs of hospitalization, medications, transfusions, and laboratory tests, among others [2, 3, 5]. Therefore, temperature monitoring is essential during surgery.

Based on these premises, it is necessary to conduct studies on hypothermia in surgical patients admitted to the intensive care unit (ICU), as hypothermia may be associated with greater complications and mortality. The results could provide the ground to develop strategies, protocols, and algorithms that minimize the occurrence and deleterious effects of hypothermia in patients undergoing major surgeries.

In this study, we aimed to describe the characteristics and outcomes of hypothermia in patients undergoing major surgeries who were admitted to the ICU.

## Materials and methods

### Ethical considerations

This study was approved by all ethics committees, such as of Medical School, University of São Paulo–São Paulo, SP, Brazil (approval number 55828016.1.2007.0068); Hospital Israelita Albert Einstein—São Paulo, SP, Brazil (approval number 55828016.1.2007.0068); Hospital de Câncer de Barretos, Pio XII Foundation–Barretos, SP, Brazil (approval number 55828016.1.2007.0068); Hospital de Base, Medical School of São José do Rio Preto—São José do Rio Preto, SP, Brazil (approval number 55828016.1.2007.0068); and Universidade Federal de Mato Grosso, Cuiabá, MT, Brazil (approval number 55828016.1.2007.0068). Written informed consent was obtained from all patients, except for two hospitals (AC Camargo Câncer Center and Hospital Sepaco) that waived the requirement for informed consent due to the observational nature of the study without direct contact with patients. The study was conducted in accordance with Resolution No. 466 of December 12, 2012, and the Declaration of Helsinki.

### Study design and patients

This prospective, observational, pre-planned secondary analysis of a multicenter study recently published [6], nationwide had a 1-month collection period, plus a 28-day follow-up to verify the complications and mortality of surgical patients who required postoperative ICU care.

Recruitment of ICUs was carried out in conjunction with the Brazilian Intensive Care Medicine Association (AMIB) through the society’s website, email campaigns, and invitation letters sent individually to each of the intensive care doctors coordinating teams in ICUs.

Patients were included from May 1, 2017, and inclusions were completed in September of the same year, with complete follow-up until November 30, 2017. Patients older than 18 years, undergoing non-cardiac surgeries, and requiring postoperative care in the ICU were included. Patients with multiple surgical reinterventions in the same hospitalization, ICU readmission in the same hospitalization of inclusion in the study, patients previously included in this study, and pregnant women were considered ineligible for the study.

All data were obtained using an electronic form (RedCap^®^), and instructions and training for the proper completion of the form were made available to researchers.

Demographic data, Simplified Acute Physiology Score (SAPS) 3, [7] Sepsis-related Organ Failure Assessment (SOFA) score [8] on admission, American Society of Anesthesiologists (ASA) physical status, chronic disease, and characteristics of the surgeries performed were collected. During the 7 days of hospitalization or discharge from the ICU (whichever occurred first), complications were collected daily in the ICU. Additionally, on the 28th day, the vital status of survivors or non-survivors throughout the ICU and hospital stay was verified.

### Outcomes

The primary outcome measure was the proportion of patients who developed postoperative hypothermia (temperature ≤ 35°C) along with its related factors. The secondary outcome was the proportion of complications associated with hypothermia during ICU stay, as described below.

Cardiovascular complications: need for vasopressors for more than 1 h despite volume resuscitation, according to a volume challenge of 1000 mL of crystalloid for up to 1 h to reach a mean arterial pressure greater than 60 mmHg. If an arterial pressure greater than 60 mmHg was not reached, a vasopressor was instilled.

Respiratory complications: acute PaO_2_/FiO_2_ < 200 in patients without previous pulmonary disease, need for reintubation, or difficulty in weaning from mechanical ventilation during the postoperative period as a result of failure in the spontaneous breathing test according to each institution’s protocols.

Renal complications: a 30% increase in creatinine, a urinal rate less than 0.5/kg/h, a renal SOFA score greater than 2, or need for dialysis during ICU stay.

Neurological complications: behavior change based on an acute fluctuation in mental status and a Richmond Agitation Sedation Scale (RASS) score of 0 within 24 h, and agitation determined by a RASS score greater than or equal to +2.

Coagulation complications: acute bleeding not justified by surgical lesions above 100 mL/h associated with the fall of three hematocrit points.

Gastrointestinal complications: acute abdominal distension, uncontrollable nausea and vomiting, or moderate to high debit of the fistula.

Infectious complications: the criteria used for identification of the foci and infectious agents were elaborated and revised according to the guidelines by the Center for Disease Control and Prevention [9–12].

Additionally, the duration of mechanical ventilation and ICU and hospital stay, as well as mortality in the ICU and hospital, were verified.

Patients within the first 24 h of ICU admission with axillary, tympanic, or esophageal temperature less than 36°C, and patients with temperatures less than 35°C were compared with patients without hypothermia (temperature greater than or equal to 36°C) in terms of demographic, clinical, and laboratory data. Possible complications related to patients with a hypothermia in the postoperative period were also analyzed. However, the central hypothesis was only tested with postoperative hypothermia defined as a temperature ≤ 35°C while seeking independent risk factors. When peripheral temperature was used, it reflected the body’s core temperature.

### Data quality assurance

The following procedures were adopted to ensure the quality of data:

1. All researchers participated in the training session before the beginning of the study to ensure consistency of the study procedures, including data collection.
2. The researchers were able to call the study coordinating center to resolve any issues or problems.
3. The Coordinating Center reviewed all screening, inclusion, follow-up, consistency, and completeness data to solve any problems with the respective participating centers.

### Statistical analysis

Considering data from the literature, we assumed a minimum hypothermia rate of 60% in patients undergoing major surgeries [3]. Therefore, we estimated that at least 500 patients would be necessary, allowing the inclusion of 20 to 30 explanatory variables (related factors) in a robust logistic regression model with hypothermia (temperature ≤ 35°C) as a dependent variable.

For the study to be representative in Brazil, the participating centers respected the regional distribution of ICUs in Brazil, according to the 2016 census conducted by AMIB, that is, approximately 55% of patients from the Southeast region, 15% in the Southeast region, 15% in the Northeast region, and 15% in the Midwest and North regions [13].

### Statistical planning

Categorical variables were presented as absolute and relative frequencies. Quantitative variables were presented as means and standard deviations or as medians and 25th-75th percentiles (Q1-Q3) when appropriate. We used the Kolmogorov–Smirnov test to evaluate the distribution patterns of continuous numerical variables.

No-hypothermic and normothermic analyses were performed. Proportions were compared using the chi-square test or Fisher’s exact test if assumptions for chi-square use were violated. Quantitative variables were compared using the Mann–Whitney test, t-test, or non-normal distribution analysis variance (ANOVA), when appropriate.

The association between explanatory variables and response was evaluated using logistic regression models (stepwise), and Kaplan–Meier curves were adjusted according to Cox regression. The variables selected in the bivariate analyses (P < 0.2) and those considered clinically relevant were subjected to multiple logistic regression analyses, and the remaining variables in this model were subjected to Kaplan–Meier adjustment according to Cox regression to verify whether hypothermia (temperature ≤35°C) was a strong predictor of postoperative complications. We first evaluated collinearity by examining the dispersion matrix and Pearson’s correlation coefficient for continuous variables, or cross-tabulation for categorical variables. We also evaluated linearity using variance inflation factor (VIF) analysis. Variables with substantial collinearity were excluded from the analysis. The analyses logistic regression results were expressed as rate ratios (RRs) and results of Cox regression analyses were expressed as hazard risks (HRs), and the respective 95% confidence intervals (CIs). All probabilities of significance (P-values) were two-tailed, and P-values were considered statistically significant at P < 0.05. Statistical Package for Social Sciences software version 26.0 (SPSS Inc., Chicago, IL, USA) was used to perform the analyses.

## Results

**Fig 1:**
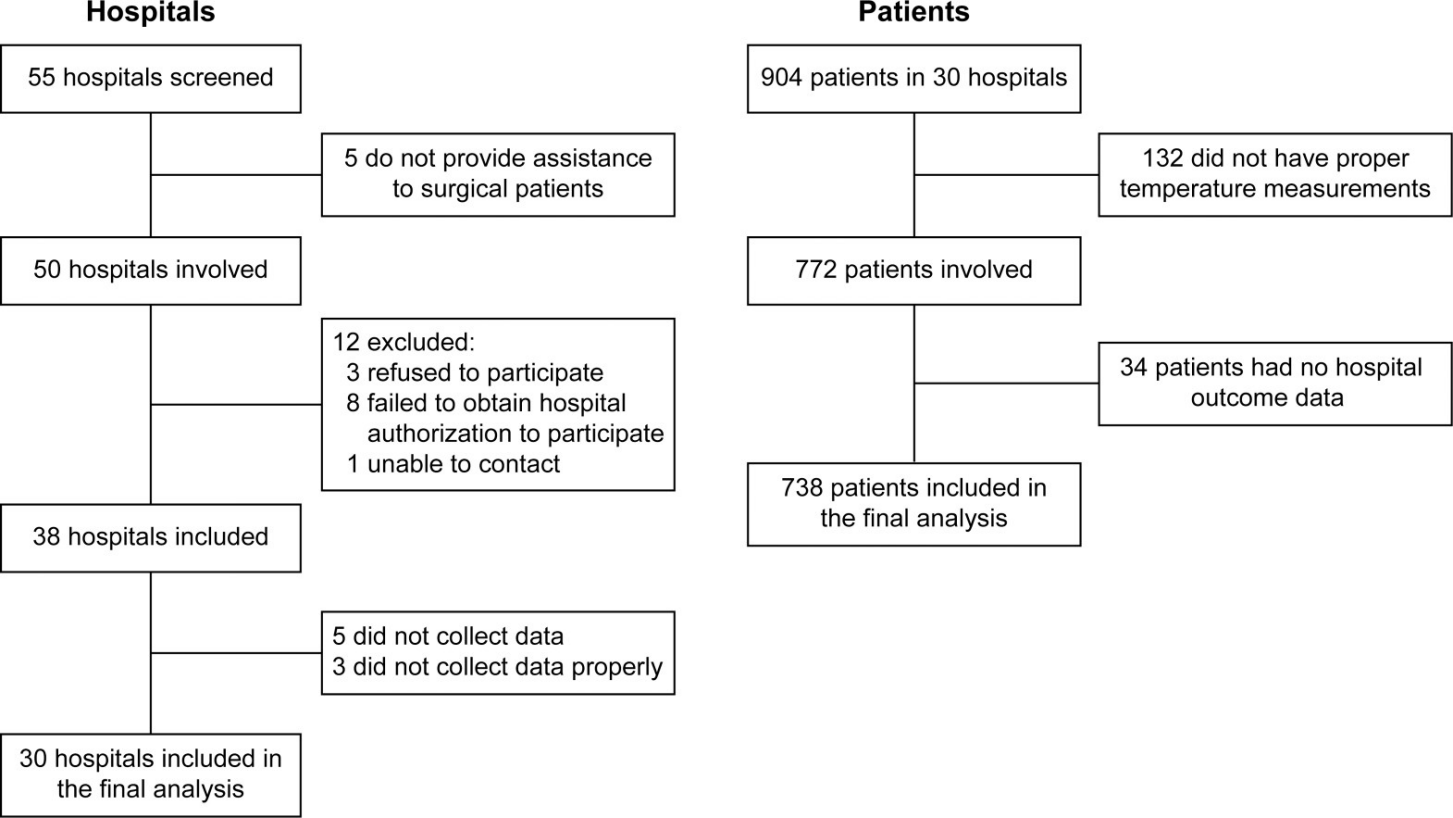
Flow chart of the study hospitals and patients.

Fifty-five eligible hospitals were selected randomly at national level, and five refused to participate. Of the 55 hospitals, 38 (70%) answered the questionnaires required to participate in the study. However, only 30 ICUs were included in the study, and for a period of 1 month, they provided data from 25,500 surgical patients, of whom 904 were indicated for postoperative intensive care and included in the analysis. Of all patients, only 738 had their temperature measured adequately on admission to the ICU (Fig 1).

Of the 738 patients included in the analysis, 48.2% were admitted to private hospitals and 51.7% were admitted to public hospitals. Of all hospitals, 59.6% were from the Southeast, 26.6% from the South, 9.3% from the Midwest, and 4.4% from the North and Northeast regions of the country. The median age (Q1- Q3) of the patients was 62 (50–72) years, the majority were men, and the median SAPS 3 was 42 (32–53). The median time of surgery was 240 (180–360) min, and elective surgeries were more prevalent (69.2%). About 80.4% of patients presented with underlying diseases, mainly hypertension, cancer, and smoking. The median duration of ICU stay was 2 (1–4) days.

The percentage of patients with temperature <35°C (median [Q1-Q3], 34.7°C [34.3–34.9°C]) was 19.1% (95% CI = 16.1–22.5) and that of patients with temperature <36°C (median [Q1-Q3], 35.4°C [35.0–35.8°C]) was 64% (95% CI = 58.3–70.0) (Table 1).

**Table 1:** Characteristics of patients with and patients without postoperative hypothermia.

Characteristic	All patients	≥36°C	<36°C	P-value	≥35°C	<35°C	P-value
**Total, n (%)**	738 (100)	266 (36)	472 (64.0)		597 (80.9)	141 (19.1)	
**Age, years (median,** Q1-Q3**)**	**62 (50–72)**	**60 (42–70)**	**65 (53–74)**	**0.000** ^**a**^	**61 (48–71)**	**69 (59.7–77.2)**	**0.000** ^**a**^
< 50 years old	181 (24.6)	86 (32.5)	95 (20.2)	0.000^b^	163 (27.4)	18 (12.8)	0.000^b^
50–59 years old	125 (17.0)	45 (17.0)	80 (17.0)	1.000^b^	108 (18.2)	17 (12.1)	0.083^b^
60–69 years old	187 (25.4)	65 (24.5)	122 (25.9)	0.675	147 (24.7)	40 (28.4)	0.3644^b^
70–79 years old	151 (20.5)	42 (15.8)	109 (23.1)	0.018^b^	110 (18.5)	41 (29.1)	0.005^b^
≥ 80 years old	92 (12.5)	27 (10.2)	65 (13.8)	0.156^b^	67 (11.3)	25 (17.7)	0.039^b^
**Men, n (%)**	370 (54.3)	130 (56)	240 (53.3)	0.502^b^	304 (55.9)	66 (47.8)	0.090^b^
**SAPS 3 (median,** Q1-Q3**)**	42 (32–53)	41 (31–53.7)	42 (32–52)	0.890^a^	40 (31–52)	45 (37–55.2)	0.002^a^
**SOFA on admission (median,** Q1-Q3**)**	2 (1–5)	2 (1–5)	2 (1–4)	0.131^a^	2 (1–5)	2 (1–5)	0.952^a^
**BMI (median,** Q1-Q3**)**	25 (22–28)	25 (22–28)	25 (22–28)	0.856^a^	25 (22–28)	25 (22–27)	0.718^a^
**Known ethnicity, n (%)**	714 (100)	257 (36)	457 (64)	0.906^b^	580 (81.2)	134 (18.8)	0.347^b^
**Caucasian**	521 (73)	187 (72.8)	334 (73.1)		427 (73.6)	94 (70.1)	
**Brown**	133 (18.6)	49 (19.1)	84 (18.4)		103 (17.8)	30 (22.4)	
**Black**	46 (6.4)	15 (5.8)	31 (6.8)		40 (6.9)	6 (4.5)	
**Other**	14 (2.0)	6 (2.3)	8 (1.8)		10 (1.7)	4 (3.0)	
**ASA score (median,** Q1-Q3**)**	2 (2–3)	2 (2–3)	2 (2–3)	0.05^a^	2 (2–3)	2 (2–3)	0.306^a^
**Duration of surgery, min (median,** Q1-Q3**)**	**240 (180–360)**	**240 (150–317.5)**	**270 (180–420)**	**0.000** ^a^	**240 (176–360)**	**240 (165–320)**	**0.177** ^**to**^
**Type of surgery, n (%)**				**0.039** ^**b**^			**0.070** ^**b**^
**Elective**	512 (69.5)	332 (70.3)	180 (67.9)		403 (67.6)	109 (77.3)	
**Urgent**	116 (15.7)	35 (13.2)	81 (17.2)		98 (16.4)	18 (12.8)	
**Type of surgery, n (%)**							
**Abdominal**	**211 (28.6)**	**63 (23.8)**	**148 (31.4)**	**0.029** ^**b**^	**163 (27.3)**	**48 (34)**	**0.114** ^**b**^
**Oncologic**	185 (25.1)	69 (26)	116 (24.6)	0.661^**b**^	146 (24.5)	39 (27.7)	0.436^**b**^
**Neurological**	**172 (23.3)**	**76 (28.7)**	**96 (20.3)**	**0.010** ^**b**^	**152 (25.5)**	**20 (14.2)**	**0.004** ^**b**^
**Orthopedic**	124 (16.8)	38 (14.3)	86 (18.2)	0.177^**b**^	100 (16.8)	24 (17)	0.945^**b**^
**Vascular**	54 (7.3)	21 (7.9)	33 (7.0)	0.641^**b**^	43 (7.2)	11 (7.8)	0.810^**b**^
**Thoracic**	41 (5.6)	14 (5.3)	27 (5.7)	0.804^**b**^	32 (5.4)	9 (6.4)	0.637^**b**^
**Urological**	36 (4.9)	10 (3.8)	26 (5.5)	0.294^**b**^	28 (4.7)	8 (5.7)	0.629^**b**^
**Head and neck**	**31 (4.2)**	**17 (6.4)**	**14 (3.0)**	**0.025** ^**b**^	**30 (5.0)**	**1 (0.7)**	**0.021** ^**b**^
**Gynecological**	15 (2.0)	3 (1.1)	12 (2.5)	0.193^**b**^	13 (2.2)	2 (1.4)	0.564^**b**^
**Other**^**c**^	47 (6.4)	18 (6.8)	29 (6.1)	0.730^**b**^	33 (5.5)	14 (9.9)	0.055 ^**b**^
**Night surgery, total n (%)**	78 (14)	22 (10.8)	56 (15.7)	0.108^**b**^	63 (14.1)	15 (13.4)	0.848 ^**b**^
**Coexisting medical conditions, n (%)**	**577 (79)**	**186 (71.3)**	**391 (83.4)**	**0.000** ^**b**^	**453 (76.8)**	**124 (88.6)**	**0.002** ^**b**^
**Systemic arterial hypertension**	**334 (45.3)**	**97 (36.6)**	**237 (50.2)**	**0.000** ^**b**^	**255 (42.8)**	**79 (56)**	**0.005** ^**b**^
**Cancer**	134 (18.2)	48 (18.1)	86 (18.2)	0.971^**b**^	104 (17.4)	30 (21.3)	0.289^**b**^
**Diabetes mellitus**	**154 (20.9)**	**43 (16.2)**	**111 (23.5)**	**0.019** ^**b**^	**116 (19.5)**	**38 (27.0)**	**0.049** ^**b**^
**Coronary insufficiency**	**47 (6.4)**	**7 (2.6)**	**40 (8.5)**	**0.002** ^**b**^	**28 (4.7)**	**19 (13.5)**	**<0.000** ^**b**^
**COPD**	**39 (5.3)**	**7 (2.6)**	**32 (6.8)**	**0.016** ^**b**^	**28 (4.7)**	**11 (7.8)**	**0.139** ^**b**^
**Other coexisting medical conditions**	217 (29.4)	80 (30.2)	137 (29)	0.740^**b**^	171 (28.7)	46 (32.6)	0.357^**b**^
**Type of anesthesia, n (%)**				0.509^**b**^			0.615^**b**^
**General**	542 (74.1)	199 (75.1)	343 (73.6)		443 (74.8)	99 (71.2)	
**Neuraxial**	67 (9.2)	20 (7.5)	47 (10.1)		54 (9.1)	13 (9.4)	
**General and neuraxial**	122 (16.7)	46 (17.4)	76 (16.3)		95 (16)	27 (19.4)	
**Type of hospital, n (%)**				0.801^**b**^			0.380^**b**^
**Public**	368 (49.9)	131 (49.2)	237 (50.2)		293 (49.1)	75 (53.2)	
**Private**	370 (50.1)	135 (50.8)	235 (49.8)		304 (50.9)	66 (46.8)	
**Intraoperative temperature control, n (%)**	**211 (28.6)**	**148 (31.4)**	**63 (23.8)**	**0.029** ^**b**^	**48 (34)**	**163 (27.3)**	**0.114** ^**b**^
**Axillary temperature control, n (%)**	185 (25.1)	69 (26)	116 (24.6)	0.661^**b**^	146 (24.5)	39 (27.7)	0.436^**b**^
**Tympanic temperature control, n (%)**	**172 (23.3)**	**76 (28.7)**	**96 (20.3)**	**0.010** ^**b**^	**152 (25.5)**	**20 (14.2)**	**0.004** ^**b**^
**Esophageal temperature control, n (%)**	124 (16.8)	38 (14.3)	86 (18.2)	0.177^**b**^	100 (16.8)	24 (17)	0.945^**b**^

Abbreviations: ASA = American Society of Anesthesiologists, BMI = body mass index, COPD = chronic obstructive pulmonary disease, ICU = intensive care unit, Q1-Q3 = 25–75 percentiles, n = number of patients, SAPS = Simplified Acute Physiology Score, SOFA = Sepsis-related Organ Failure Assessment.

^a^Mann–Whitney U test.

^b^Chi-square test.

^c^Other surgeries were plastic and ophthalmology.

Patients with hypothermia were older, had undergone abdominal surgeries, had undergone procedures of longer duration, had more comorbidities, and stayed longer stay in the ICU and hospital (Tables 1 and 2). Patients with hypothermia after surgery presented more complications, mainly hematological and infectious complications, and stayed longer in the ICU and hospital, but required fewer vasopressors and less mechanical ventilation in the postoperative period (Table 2).

**Table 2:** Clinical outcomes in patients with and patients without (severe) postoperative hypothermia.

Features	All patients	≥36°C	<36°C	P-value	≥35°C	<35°C	P-value
**Composite Complications, n (%)**	**287 (38.9)**	**91 (34.2)**	**196 (41.6)**	**0.048** ^b^	**187 (31.4)**	**100 (70.9)**	**0.000** ^b^
**Cardiac**	116 (16.4)	47 (18.1)	69 (15.4)	0.349 ^b^	95 (16.6)	21 (15.6)	0.760 ^b^
**Respiratory**	60 (8.1)	28 (10.5)	32 (6.8)	0.074 ^b^	49 (8.2)	11 (7.8)	0.874 ^b^
**Renal**	127 (17.2)	55 (20.7)	72 (15.3)	0.061 ^b^	103 (17.3)	24 (17)	0.948 ^b^
**Hematological**	**68 (92)**	**6 (2.3)**	**62 (13.1)**	**0.000** ^b^	**23 (3.9)**	**45 (31.9)**	**0.000** ^b^
**Infection**	**67 (9.1)**	**3 (1.1)**	**64 (13.6)**	**0.000** ^b^	**14 (2.3)**	**53 (37.6)**	**0.000** ^b^
**Gastrointestinal**	56 (7.6)	17 (6.4)	39 (8.3)	0.357 ^b^	40 (6.7)	16 (11.3)	0.061 ^b^
**Reoperations, n (%)**	27 (3.7)	12 (4.5)	15 (3.2)	0.354 ^b^	23 (3.9)	4 (2.8)	0.563 ^b^
**Use of vasopressor, n (%)**	131 (17.8)	65 (24.4)	66 (14)	0.000 ^b^	109 (18.3)	22 (15.6)	0.458 ^b^
**Use of mechanical Ventilation, n (%)**	119 (16.9)	57 (22.1)	62 (13.9)	0.005 ^b^	98 (17.2)	21 (15.7)	0.667 ^b^
**Hemodialysis, n (%)**	18 (2.7)	8 (3.2)	10 (2.3)	0.501 ^b^	16 (2.9)	2 (1.6)	0.404 ^b^
**Length of ICU stay (days), median (Q1-Q3)**	2.0 (1.0–4.0)	2.0 (1.0–3.9)	2.1 (1.1–4.6)	0.045^a^	2.0 (1.0–4.0)	2.0 (1.0–4.0)	0.479^a^
**Length of hospital stay (days), median (Q1-Q3)**	9.5 (5.4–18.6)	8.4 (4.7–16.6)	9.8 (5.3–20.1)	0.033^a^	8.6 (4.9–18.4)	8.5 (4.6–14.4)	0.133^a^
**ICU mortality, n (%)**	35 (5.0)	13 (5.1)	22 (4.9)	0.907 ^b^	28 (4.9)	7 (5.3)	0.864^b^
**Hospital mortality, n (%)**	64 (9.1)	23 (9.1)	41 (9.2)	0.959^b^	51 (9.0)	13 (9.6)	0.822^b^
**Mortality at 28 days, n (%)**	60 (10.2)	22 (10.2)	38 (10.2)	0.999^b^	47 (10.0)	13 (10.9)	0.766^b^

Abbreviations: ICU = intensive care unit, Q1-Q3 = 25th-75th percentiles, n = number of patients.

^a^Mann–Whitney U test

^b^Chi-square test.

**Fig 2:**
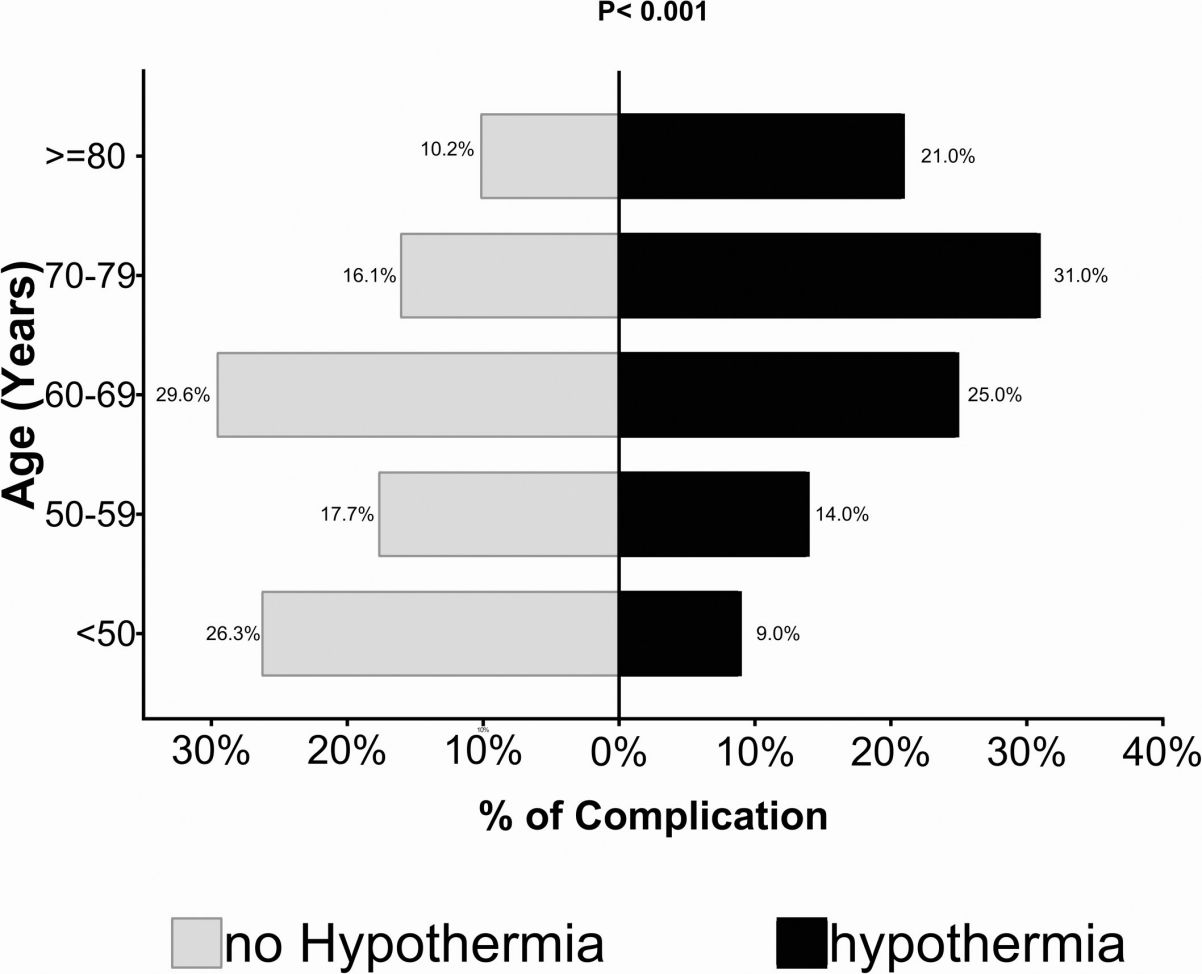
Stratification of non-hypothermia and hypothermia groups in relation to age comparing complication rates.

After stratification of the non-hypothermia and hypothermia groups in relation to age, it was found differences in complication rates (P<0.001). Older patients with hypothermia had more complications (Fig 2).

Older age (RR = 1.01, 95% CI = 1.004–1.035), longer surgical procedures (RR = 1.002, 95% CI = 1.001–1.004), more comorbidities (RR = 1.683, 95% CI = 1.016–2.788), and abdominal surgeries (RR = 1.876, 95% CI = 1.137–3.096) were independently associated with postoperative hypothermia (temperature **≤**35°C) (Table 3).

**Table 3:** Univariate and multivariate analyses of factors related to a postoperative temperature of ≤35°C.

	Univariate			Multivariate		
	RR	95% CI	P-value	RR	95% CI	P-value
**Male sex**	0.897	0.652–1.233	0.502			
**Age (per year)**	**1.002**	**1.009–1.027**	**<0.001**	**1.019**	**1.004–1.035**	**0.015**
**< 50 years old**	Reference			Reference		
**50–59 years old**	1.425	0.704–2.888	0.325	1.181	0.511–2.727	0.697
**60–69 years old**	2.464	1.353–4.487	0.003	1.678	0.809–3480	0.165
**70–79 years old**	3.375	1.844–6.179	<0.001	2.033	0.942–4386	0.071
**≥ 80 years old**	3.379	1.730–6.599	<0.001	2.851	1.230–6.607	0.015
**BMI (per kg/m** ^**2**^ **)**	0.995	0.962–1.031	0.856			
**SAPS 3 (per unit)**	1.001	0.991–1.012	0.890			
**SOFA on admission (per unit)**	0.943	0.899–0.988	0.131	0.960	0.896–1.029	0.252
**ASA score (per unit)**	1.192	0.981–1.448	0.057	1.156	0.886–1.510	0.286
**Type of surgery**			0.039			
**Elective**	Reference			Reference		
**Urgency**	1.255	0.811–1.940	0.308	0.938	0.568–1.549	0.802
**Night surgery**	1.536	0.907–2.600	0.108	1.402	0.753–2.608	0.287
**Intraoperative temperature control**	0.824	0.606–1.121	0.029	1.006	0.665–1.522	0.979
**Surgery time (per min)**	**1.003**	**1.002–1.004**	**<0.001**	**1.002**	**1.001–1.004**	**0.013**
**Type of surgery**						
**Abdominal**	**1.465**	**1.039–2.064**	**0.029**	**1.876**	**1.137–3.096**	**0.014**
**Oncological**	0.926	0.655–1.307	0.661			
**Neurological**	0.635	0.448–0.899	0.010	0.750	0.432–1.300	0.305
**Orthopedic**	1.331	0.878–2.017	0.177	0.988	0.554–1.762	0.968
**Vascular**	0.872	0.494–4.543	0.641			
**Chest**	1.088	0.560–2.113	0.804			
**Urologic**	1.487	0.705–3.132	0.294			
**Head and neck**	0.446	0.216–0.970	0.025	0.561	0.227–1.391	0.212
**Gynecologic**	2.278	0.637–8147	0.193	2.876	0.589–14.032	0.191
**Other surgeries**	0.898	0.489–1.651	0.730			
**Coexisting medical conditions**	**2.021**	**1.408–2.903**	**0.000**	**1.683**	**1.016–2.788**	**0.043**
**Systemic arterial hypertension**	1.747	1.283–2.377	0.000	1.520	1.083–2.234	0.015
**Cancer**	1.007	0.682–1.488	0.971			
**Diabetes mellitus**	1.587	1.075–2.344	0.019	1.175	0.763–1.810	0.465
**Coronary insufficiency**	3.413	1.507–7.731	0.002	2.666	1.158–6.137	0.021
**COPD**	2.681	1.166–6.160	0.016	2.155	0.922–5.038	0.76
**Other coexisting medical conditions**	0.946	0.681–1314	0.740			
**Type of anesthesia**						
**General**	1.043	0.695–1.565	0.838			
**Neuraxial**	1.422	0.751–2.694	0.280			
**General and neuroaxial**	0.950	0.639–1.438	0.838			
**Type of hospital**						
**Public**	1.039	0.770–1.404	0.802	1.060	0.713–1.576	0.774
**Private**	Reference			Reference		

Abbreviations: ASA = American Society of Anesthesiologists, BMI = body mass index, CI = confidence interval, COPD = chronic obstructive pulmonary disease, RR = rate ratio.

**Fig 3:**
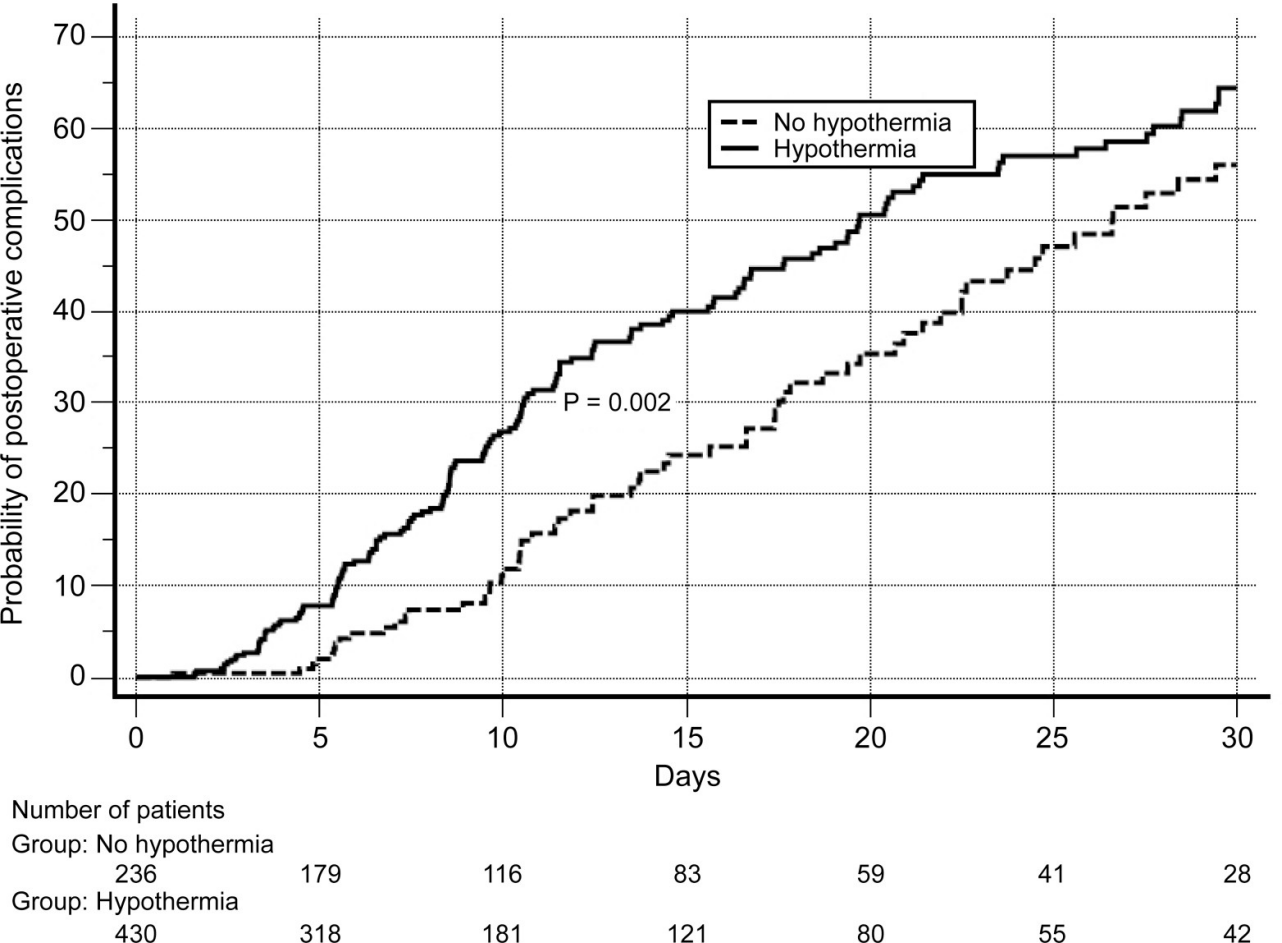
Kaplan–Meier curve of complications and hypothermia. Data were adjusted for age, comorbidities, abdominal surgery, and duration of surgery.

Patients who presented with a temperature ≤35°C in the postoperative period had a high risk for postoperative composite complications (HR = 1.523, 95% CI = 1.15–2.0) after adjusting for comorbidities, age, abdominal surgeries, and duration of surgery using a Cox regression model (Fig 3).

## Discussion

In this prospective study of 738 consecutive patients who received postoperative care in ICUs, more than half of the patients had hypothermia in the first 24 h after surgery. This is consistent with our first hypothesis that inadvertent hypothermia is common in patients in the postoperative period of major surgeries. In addition, we found that age, previous comorbidities, duration of surgery, and abdominal surgeries were strong factors for hypothermia. In our second hypothesis, multivariate analysis revealed that hypothermia in the first 24 h after surgery was associated with surgical complications, especially coagulation and infection.

The incidence of 60% of patients with temperatures below 36°C and 19% with temperatures below 35°C is compatible with those reported in several other comparable studies [4, 14, 15]. Kongsayreepong et al. [16] found that 57% of the 194 surgical patients in the ICU had a tympanic temperature below 36°C, and 28% had a temperature below 35°C. Abelha et al. [17] found that 58% of 185 surgical patients in the ICU had a tympanic temperature below 35°C, which is also comparable to the incidence in our study.

An explanation for this high hypothermia rate may be the low adherence to more aggressive intraoperative heating in hospitals in Brazil, which has been reported [15]. We found that the incidence of hypothermia after abdominal surgery was approximately 30%. This is similar to the study in cardiac surgery by Insler et al. [18], who studied more than 5,000 patients undergoing cardiac surgery and found that 28% of them had hypothermia (temperature below 36°C) in the ICU. One study [1] in patients undergoing non-cardiac surgery also showed a high incidence of hypothermia in abdominal surgeries.

We found that older patients, patients with more comorbidities, and patients who had undergone longer surgeries were more likely to have hypothermia in the ICU. These associations have been reported in the literature [4, 14, 19], indicating that these patients have unfavorable outcomes [20, 21].

In the Cox regression model, hypothermia (temperature ≤35°C) was associated with more complications regardless of age, comorbidities, time of surgery, and abdominal surgeries. Our multivariate analysis indicated that hypothermia in the ICU had a HR of 1.5 for composite postoperative complications. Insler et al. [18] and Karalapillai et al. [1] also found a strong association between inadvertent hypothermia and worse outcomes in surgical patients; however, previous studies have not demonstrated this association [16, 17]. Nevertheless, the main complications found in the present study were related to coagulation and infection. The others were not relevant, and we did not identify a higher incidence of mortality.

A decrease of 1–2°C in core temperature causes reversible damage to platelet aggregation via reduction of thromboxane A3 and has a negative impact on platelet plug formation [22]. It also damages the enzymatic system of the coagulation cascade, decreasing the formation of clots. These two combined mechanisms result in increased blood loss and the need for blood transfusion in up to 20% of cases [23, 24].

From an infectious point of view, there are at least three mechanisms that explain the influence of hypothermia on the immune system: reduction of perfusion in the tissue of the surgical incision, which hinders local access to defense cells, a decrease in the motility of macrophages, and a reduction in scar tissue formation, which increases the chance of dehiscence and secondary infection [25]. A randomized study of 200 patients undergoing colorectal surgery showed that normothermic patients had lower rates of surgical wound infection in the order of 6% compared to those maintained up to 2°C (mild hypothermia) [10].

Therefore, given these findings, we were able to demonstrate that in this large multicenter study on inadvertent hypothermia in the mixed surgical population of ICU patients, except in cardiac surgery, there is an association between hypothermia and complications, and we also verified possible factors related to this problem. Although other studies [1, 14] previously reported risk factors for hypothermia in surgery, they were from retrospective analyzes [4, 14, 19] and failed to find a correlation with postoperative complications [15, 19].

### Limitations

The limitations of our study include the observational design, which did not include cardiac surgeries and non-ICU patients, but it was a national study with participation of representative ICUs across Brazil. This factor can limit the generalization of our results and impose a direct association between cause and effect, which does not mean that the data are unimportant, because they can drive quality improvement in the institutions. Nevertheless, it is important to generate robust hypotheses. In addition, previous studies [14, 16, 17] used infrared tympanic thermometers to measure the temperature of patients, whereas we used the whole axillary, tympanic, or esophageal method, which can generate bias in the measurement of temperature due to possible underestimation of the temperature. On the other hand, it strengthens the findings as it is closer to the practice of this type of care since the study was a pragmatic analysis. Additionally, if the major bias reported is applied to our data, the hypothermia rate will be even higher. However, the balance between accuracy, safety, and cost among non-invasive thermometers is unclear. The body core temperature is defined within a narrow range, and there are differences between oral, rectal, and axillary measurements [26]. We also emphasize that in order to avoid this bias, the outcome analysis was performed in patients with a temperature ≤ 35°C, a measurement that can reflect 36°C of core temperature [27].

Another limitation of our study was related to the deficiency in the collected data. The database did not include factors that may contribute to the risk of intraoperative hypothermia, such as the surgical team and intra- and postoperative warm-up. In addition, the database recorded only the lowest temperature in the first 24 h after ICU admission, but not during the 24 h in which the lowest temperature occurred. Although, given the nature and limitations of this study, we cannot draw conclusions about the cause. Further studies are needed to determine the most appropriate way to measure the temperature of patients in ICUs and whether active heating decreases complications and mortality. Still, considering the available evidence of adverse outcomes associated with perioperative hypothermia, we suggest that all members of the healthcare team involved in the perioperative period routinely and actively keep all surgical patients warm with adequate resources.

## Conclusions

Inadvertent hypothermia in postoperative ICU patients was common, and was more prevalent after abdominal surgery, longer procedures, elderly patients, and in patients with a higher number of comorbidities. Postoperative low temperature was associated with postoperative complications, specifically coagulation and infection. Therefore, the surgical team should remain vigilant in warming patients and minimize heat loss during and after surgery.

## Supporting information

S1 Data (XLSX)
